# Hydrogen Embrittlement of the Additively Manufactured High-Strength X3NiCoMoTi 18-9-5 Maraging Steel

**DOI:** 10.3390/ma14175073

**Published:** 2021-09-04

**Authors:** Angelina Strakosova, Michaela Roudnická, Ondřej Ekrt, Dalibor Vojtěch, Alena Michalcová

**Affiliations:** 1 Department of Metals and Corrosion Engineering, University of Chemistry and Technology, Prague, Technická 5, 166 28 Prague 6, Czech Republic; michaela.fousova@vscht.cz (M.R.); Dalibor.Vojtech@vscht.cz (D.V.); Alena.Michalcova@vscht.cz (A.M.); 2 Institute of Physics, Czech Academy of Science, Na Slovance 1999/2, 182 21 Prague, Czech Republic; ekrt@fzu.cz

**Keywords:** maraging steel, selective laser melting, hydrogen embrittlement, hydrogen-induced cracking

## Abstract

The main aim of this study was to determine the susceptibility of the additively manufactured high strength X3NiCoMoTi 18-9-5 maraging steel to hydrogen embrittlement. For this purpose, samples produced by selective laser melting technology, before and after heat treatment, were used. The examined samples were electrochemically charged with hydrogen in NaCl + NH_4_SCN solution at a current density of 50 mA/cm^2^ for 24 h. The H content increased from about 1 to 15 ppm. Heat treatment did not affect the amount of H trapped in the maraging steel. Tensile testing revealed that the tensile strength of the H-charged samples was much lower than that of the uncharged samples. Moreover, the material became brittle after charging compared to the ductile as-printed and heat-treated samples with elongation values of 7% and 2%, respectively. The loss of plasticity was confirmed by fractography, which revealed transformation of the fracture surface morphology from dimple-like in the as-produced state to a brittle one with smooth facets in the H-charged state.

## 1. Introduction

Selective laser melting (SLM) technology is a modern and well-known additive manufacturing (AM) technology. The main goal of this method is to build three-dimensional products with precisely specified sizes and shapes by the successive melting of the metal powder, layer-by-layer [1,2]. SLM is a popular method used in material production because of: (i) low material consumption; (ii) defined product shapes and sizes; and (iii) produces different types of alloys, from Ti-alloys [2,3] and Mg-alloys [4,5] to ultra-high-strength maraging steels [6,7,8,9,10,11].

X3NiCoMoTi 18-9-5 maraging steel is an almost carbon-free high-alloyed steel, which is characterized by outstanding mechanical properties, such as ultimate tensile strength, hardness, high ductility, good machinability, and easy weldability [6,11,12,13]. Two-stage “solution annealing” and “aging” heat treatment contribute toward obtaining these properties. After the first heat treatment step, the steel is relatively soft with a hardness of around 30 HRC [14]. Aging is the main step involves in heat treatment, which is responsible for precipitation hardening of the material. Different types of precipitates, which form in maraging steel, were described in other research works: Ni_3_(Ti, Mo) [13], Ni_3_X (X = Ti, Al, Mo) [15], (Fe, Ni, Co)_3_(Ti, Mo) and (Fe, Ni, Co)_7_Mo_6_ [16]. Maraging steel is primarily used in military and aerospace industries and in nuclear power [14]. This critical area of application makes the steel very sensitive to hydrogen embrittlement [14,17,18,19].

Hydrogen embrittlement (HE) is manifested by a reduction in the strength and ductility of the material, which can lead to damaging the integrity of the material [17]. In work [20], the authors summarized the occurrence of the hydrogen embrittlement in materialso into two groups: (i) internal—occurs due to the H trapped in the material during production; (ii) external—occurs due to the material exposure to a hydrogen-containing environment. In contrast, N. Eliaz et al. [21] describes the HE of materials due to: (i) hydrogen-induced cracking (HIC); (ii) stress corrosion cracking (SCC); and (iii) hydrogen em brittlement (HE). It is known that maraging steel is prone to hydrogen embrittlement due to service environment exposure [18,22]. Dislocations, grain boundaries, and interstitial site precipitates, etc., are the main hydrogen traps in these steels [17,19,22,23,24]. After the hydrogen is trapped in the material, it causes material cracking and destruction [25].

Studies [17,18,19,22,25,26,27] describe the HE of the conventional maraging steels, while in other works [28,29], the HE of the additively manufactured ones is investigated. Different hydrogen charging (HC) conditions of the materials were used in each work. The main difference in the HE of the two above-mentioned types of steel involves the fracture mechanism. In the conventional one, cracks propagate intergranularly, while a quasi-cleavage fracture appears in the additively manufactured one. As for the mechanical properties and hydrogen content, the measured values vary greatly depending on the hydrogen charging medium, current density, and charging duration [17,18,19,22,25,26,27,28,29]. 

It is thus important to thoroughly study how the hydrogen charging affects the behavior of maraging steel when produced by selective laser melting technology. The study aimed to discover how the tensile properties and fracture surfaces of the previously studied X3NiCoMoTi 18-9-5 maraging steel produced by SLM [13] changed after the hydrogen charging, using the same conditions as in the work by [28]. While in work [28], only the HE of the as-fabricated state is described, here, we compare the differences between the HE of the as-printed and heat-treated steel.

## 2. Materials and Methods

**Figure 1:**
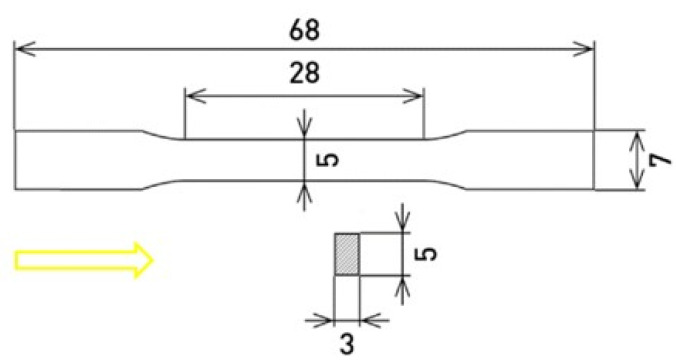
Dimensions of the studied samples (units: mm).

SLM solution 280 HL (NETME Center, Brno, Czech Republic) was used to produce samples of the X3NiCoMoTi 18-9-5 maraging steel in a dog bone shape (Figure 1), from the powder; characteristics are described in detail in the previous paper [13]. Samples were purchased from the NETME Center. The building directions of the samples (marked with a yellow arrow) was oriented parallel to their longitudinal axis. The porosity of the studied material was measured using ImageJ software. For heat treatment, electric resistance furnaces were used. Half of the 12 samples were heat-treated (HT) under the regime consisting of solution-annealing at 820 °C for 1 h, followed by aging treatment at 490 °C for 6 h. Both stages ended with air-cooling. Microstructure characterization was studied on metallographic samples using a scanning electron microscope (SEM; TESCAN VEGA 3 LMU, Brno, Czech Republic). To produce metallographic samples, several steps were involved: (i) grinding on SiC abrasive papers (P280–P4000); (ii) polishing on a diamond paste (D 2 μm); (iii) final polishing using the suspension Eposil F. To make the microstructure observable, the Nital 2 solution (2 mL HNO_3_ + 98 mL ethanol) was used. For the detailed microstructure study, a transmission electron microscope (TEM; JEOL 2200 FS, Akishima, Japan equipped by EDS-OXFORD Instruments, High Wycombe, UK) was used. For this purpose, cutting and grinding (P4000) operations were used to make very thin plates with a diameter of 3 mm. The next step was ion polishing in an Ar atmosphere using the PIPs GATAN machine.

An electrochemical method was used for the H-charging of the studied maraging steel. Samples (three of each state) of the studied maraging steel were electrochemically charged by H in the distilled water solution containing 3 wt.% NaCl and 0.3 wt.% NH_4_SCN [28,29]. A sample was connected as the cathode and a Pt coil as the anode. H-charging took 24 h under the following conditions: (i) constant current density of 50 mA/cm^2^; (ii) room temperature. Immediately after the H-charging process was completed, the tensile tests with a strain rate of 10^−5^ s^−1^ were performed (universal loading machine LabTest 5.250SP1-VM) according to the CSN EN ISO 6892-1 standard. Tensile load acted in the building direction of the samples. Tests were carried out at room temperature. Further, a part of the broken sample was used to document the fracture surface using SEM. The other sample part was used to measure the H content. The precision cut-off BRILLANT 220 (ATM GmbH, Mammelzen, Germany) machine was used to cut each studied sample into two small pieces, suitable for the elemental combustion analysis. Between the cutting operation and the H-content measurements, all specimens stayed in the liquid N. 

Two measurements of the H-content were conducted for each sample. For this purpose, a Bruker Galileo 8 analyzer (Kalkar, Germany) was used. This analyzer was based on the inert gas fusion (IGF) principle. It involved the fusion (melting) of the sample material in a steam of inert gas (5.0 nitrogen) in a graphite crucible at temperatures up to 2500 °C. Hydrogen was released in the molecular form during the analysis. The gas flow passed through reagents and molecular sieves, after which the gas composition was exclusively H_2_ and a carrier gas. The gas steam was directed to the two-way precise thermo-stabilized thermal conductivity cell (TCD) with a resolution of ~0.01 ppm. During the analysis, signals from two-way TCD detector were compared [30]. 

## 3. Results and Discussion

**Figure 2:**
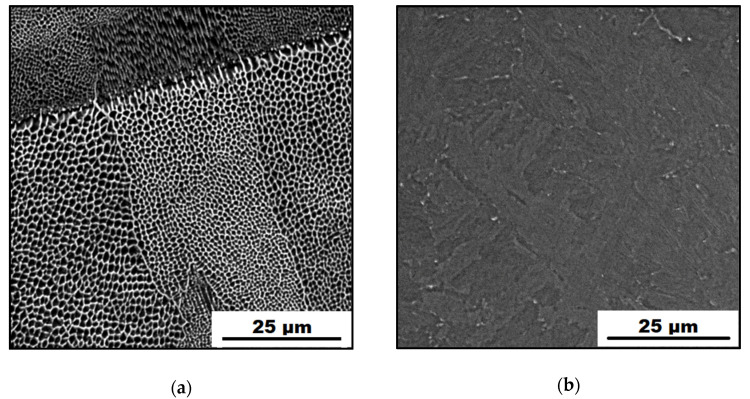
SEM micrographs of the studied maraging steel: (**a**) as-printed; (**b**) heat-treated.

The microstructure of the investigated X3NiCoMoTi 18-9-5 maraging steel before H-charging is shown in Figure 2. Studied samples, which were produced by selective laser melting technology, showed an average density of 99.7%. Specimens, which were used for microstructure observation, were prepared perpendicular to the building direction.

Figure 2a shows a SEM micrograph of the as-printed sample. It is clearly visible that it is characterized by a fine cellular microstructure. Cells of an average diameter of 1 μm are characteristic structural components resulting from high cooling rates (up to 10^6^ K/s) during the additive manufacturing production [31]. The SEM micrograph of the HT samples is shown in Figure 2b. It can be observed that solution-annealing and aging treatment had a huge impact on the maraging steel microstructure. Cell boundaries disappeared; microstructure homogenized and coarsened. Moreover, heat treatment contributed to changes in the phase composition of the material. In the previous study [13], it was found that the as-printed material consisted of 94% α-phase (martensite) and 6% γ-phase (retained austenite). In contrast with this, HT material became fully martensitic with 100% of the α-phase. As known, the aging treatment of the X3NiCoMoTi 18-9-5 maraging steel at lower temperatures is responsible for the precipitation hardening and has almost no effect on the microstructure changes observable under SEM resolution [13].

The microstructure analysis of the hydrogen-charged samples was also performed for uncharged ones. The microstructure remained the same so that only images of the original microstructure are presented. This means that the absorbed hydrogen atoms do not change the phase composition of the studied alloy, which happens with Ti alloys [32].

**Figure 3:**
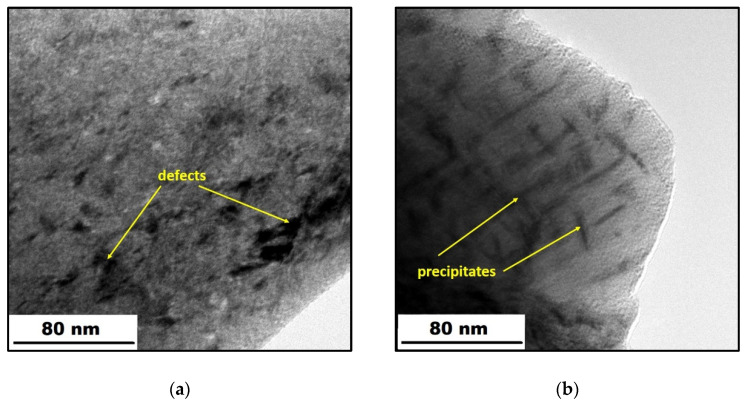
TEM micrographs of the X3NiCoMoTi 18-9-5 steel: (**a**) as-printed; (**b**) heat-treated.

The TEM micrographs, which depict the effect of heat treatment on the microstructure, are shown in Figure 3. In Figure 3a, one can observe light areas representing the primary alloy, and dark ones being the defects, mostly dislocations, which are formed due to very high cooling rates during SLM [29]. In contrast, the dark rods in Figure 3b correspond to homogeneously distributed precipitates that occurred due to heat treatment application. In work [13], it was found that these precipitates are intermetallic phases of Ni_3_(Ti, Mo).

**Figure 4:**
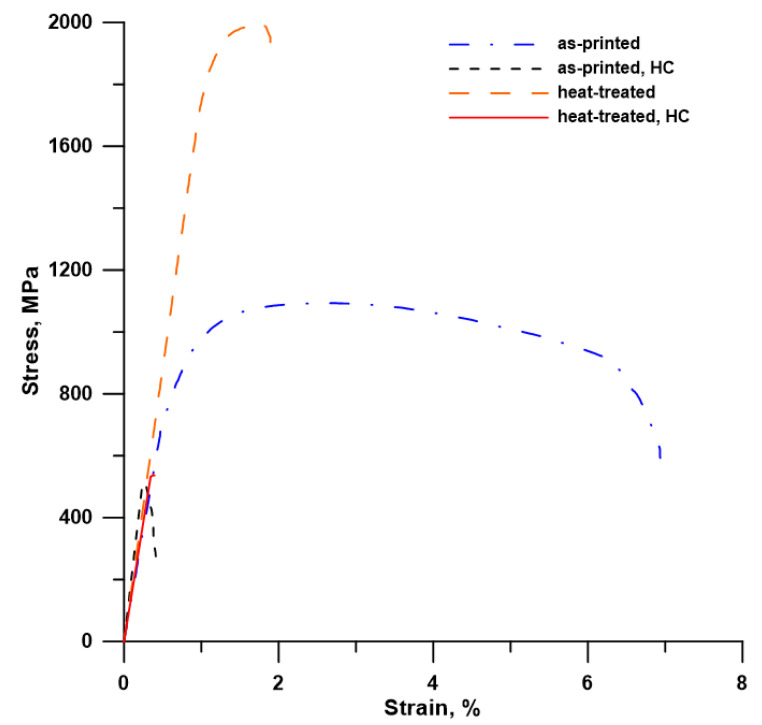
Tensile stress–strain curves of the maraging steel: effect of heat treatment and hydrogen charging (HC).

To reveal the impact of H-charging on the mechanical properties of the investigated maraging steel, tensile tests were conducted. All samples were tested parallel with the printing direction. Stress–strain curves in Figure 4 clearly show a transition from ductile to brittle behavior after H-charging. H-charging of the samples had a tremendous effect on both ductility and ultimate tensile strength (UTS). Both H-charged (HC) materials (as-printed and heat-treated) showed no elongation and yield strength (TYS0.2) and had a similar value of UTS (Table 1). The reduction in the UTS-values was huge, especially in the case of heat-treated samples, the strength of which was significantly increased by the applied heat treatment compared to the as-printed samples. A sharp increase in TYS and UTS, accompanied by a remarkable reduction in elongation (from 6.9 to 1.9%), can be explained by the precipitation strengthening of the maraging steel by very fine Ni_3_(Ti, Mo) precipitates homogeneously distributed in the material volume (Figure 3b) [13]. The reduced elongation can also be ascribed to the fact that HT samples were fully martensitic compared to the as-printed ones, having 6% of the relatively soft and ductile γ-phase. After H-charging, the UTS values decreased by 55% and by 73% in the as-printed and heat-treated samples, respectively. 

The observed changes in mechanical properties indicate a strong susceptibility of the maraging steel produced by SLM to hydrogen embrittlement because of its defective structure. In the case of the as-printed material, pores, vacancies, and high density of dislocations are responsible for the absorption, transportation, and trapping of hydrogen atoms [24]. The phase composition of the maraging steel also has some impact on the hydrogen atoms trapping. In works [19,25], the authors claimed that retained austenite has a higher hydrogen solubility than martensite. It means that the γ-phase is another hydrogen-trapping place. Regarding this, places, where cracks appear and propagate in the maraging steel, are concentrated along the grain boundaries. In the case of the heat-treated samples, precipitate boundaries become other trapping sites for H atoms, besides the above-mentioned defects [29]. When the hydrogen atoms are trapped into the trapping sites, they start to generate stresses in the material matrix, yielding formation and propagation of cracks in the material volume.

**Figure 5:**
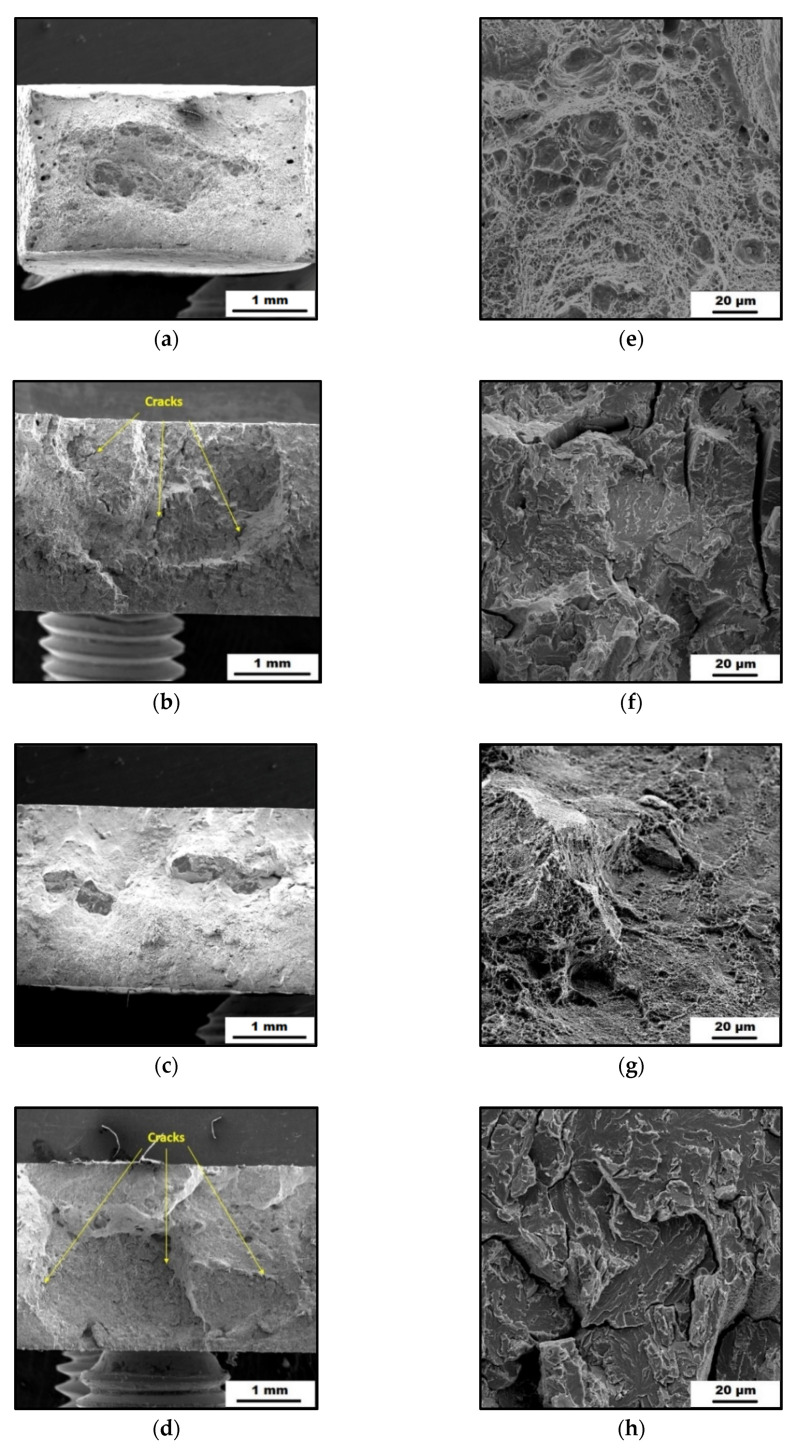
SEM micrographs (**a**–**d**) and detailed SEM micrographs (**e**–**h**) of the fracture surfaces of the maraging steel: (**a**,**e**) As-printed; (**b**,**f**) As-printed, HC; (**c**,**g**) Heat-treated; (**d**,**h**) Heat-treated, HC.

The SEM micrographs of the investigated fracture surfaces are shown in Figure 5. The fracture surface of the as-printed samples (Figure 5a,e) showed a dimple-like morphology characteristic for ductile fracture. Moreover, it can be observed that the SLM samples have residual porosity located mainly near the sample surface. The fracture surface of the heat-treated samples (Figure 5c,g) looks a bit different, even though the dimple-like morphology still predominates. The plastic deformation during fracture of the heat-treated sample is reduced as compared to the as-printed one, which is consistent with the observed difference in elongation (Figure 4, Table 1).

The effect of H-charging on the loss of ductility is clearly visible in Figure 5f,h. The ductile dimple-like surfaces changed to brittle, characterized by cleavage planes. Moreover, cracks perpendicular to the loading direction that appeared as a result of the H-charging process and subsequent tensile testing can be observed on the fracture surfaces (Figure 5b,d).

Crack formation and propagation were caused by the fact that the studied material was exposed to hydrogen charging with a current density of 50 mA/cm^2^ for 24 h. During this time, the hydrogen atoms were absorbed on the surface of the material, and then they were transported and trapped by the defective places of the microstructure. In the as-printed samples, these were mainly vacancies, dislocation sites, pores, and some percentage of the retained austenite (γ-phase). In the heat-treated material, which did not contain any γ-phase, precipitate boundaries were added as other hydrogen trapping sites. 

The main mechanism of crack formation can be described as follows: when a large number of hydrogen atoms were accumulated in trapping sites, it caused large internal stresses in the crystal lattice of the material and weakening of atomic forces between atoms of the studied steel. That supported the initiation and promotion of crack growth during loading [29].

**Figure 6:**
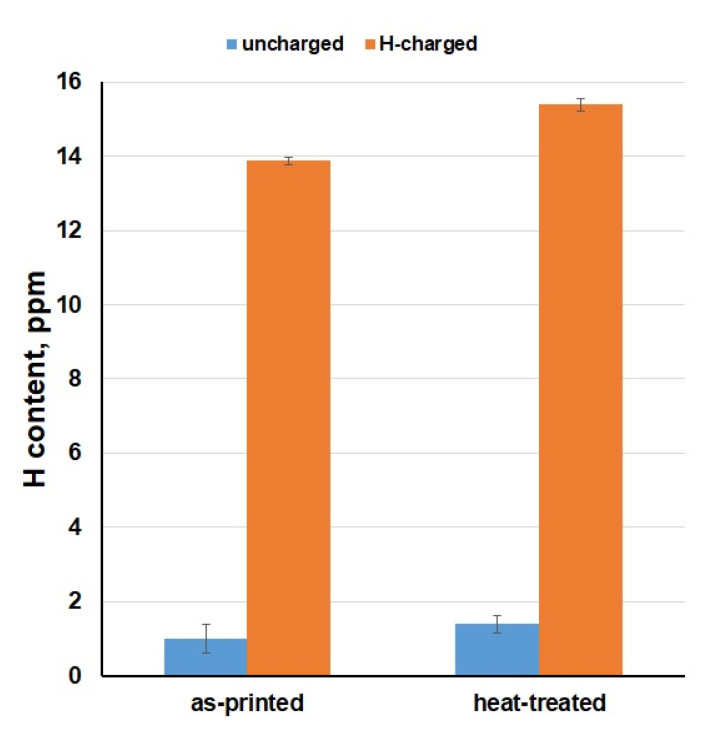
H content in the studied X3NiCoMoTi 18-9-5 steel.

Combustion analysis enabled to measure the amount of hydrogen absorbed to the material volume (Figure 6), which caused a reduction of mechanical properties and damaged the material integrity. It can be observed that uncharged samples had about 1.0–1.5 ppm of the hydrogen. In contrast, H content increased up to 14–15 ppm in the HC samples. In addition, measured H amounts demonstrated a slightly higher susceptibility to hydrogen embrittlement for the heat-treated state of the material. The obtained results clearly show us that the X3NiCoMoTi 18-9-5 maraging steel produced by SLM is very susceptible to hydrogen embrittlement due to the content of numerous crystallographic defects.

## 4. Conclusions

In this work, the effect of hydrogen charging on the tensile properties of the high-strength X3NiCoMoTi 18-9-5 maraging steel prepared by SLM was studied in dependence on the applied heat treatment. It was found that the investigated steel is very prone to hydrogen embrittlement. After saturation, UTS of the as-printed and heat-treated samples decreased from 1093 to 511 MPa and from 1996 to 539 MPa, respectively. Moreover, the material became completely brittle. Combustion analysis showed that the hydrogen content increased from about 1.0–1.5 ppm to about 14–15 ppm and was comparable for all samples, both as-printed and heat-treated. Based on the obtained results, we can conclude that the SLM-produced maraging steel shows a high degree of susceptibility to hydrogen embrittlement, regardless of the applied heat treatment. 

## Figures and Tables

**Table 1:** Selected tensile properties before and after H-charging.

Sample	TYS0.2, MPa	UTS, MPa	A, %
As-printed	926.5 ± 9.3	1107.3 ± 10.7	9.8 ± 1.8
As-printed, HC	-	628.2 ± 7.5	-
Heat-treated	1939.7 ± 2.4	1974.9 ± 14.5	2.5 ± 0.3
Heat-treated, HC	-	674 ± 8.3	-

## Data Availability

Data is contained within the article.
